# The Analysis of Risk Factors in the Conversion from Laparoscopic to Open Cholecystectomy

**DOI:** 10.3390/ijerph17207571

**Published:** 2020-10-18

**Authors:** Łukasz Warchałowski, Edyta Łuszczki, Anna Bartosiewicz, Katarzyna Dereń, Marta Warchałowska, Łukasz Oleksy, Artur Stolarczyk, Robert Podlasek

**Affiliations:** 1 Department of General Surgery, Clinical Regional Hospital No. 2 in Rzeszów, 35-301 Rzeszów, Poland; podlasek.robert@gmail.com; 2 Institute of Health Sciences, Medical College of Rzeszów University, 35-959 Rzeszów, Poland; eluszczki@ur.edu.pl (E.Ł.); abartosiewicz@ur.edu.pl (A.B.); kderen@ur.edu.pl (K.D.); 3 NZOZ Primadent in Rzeszów, 35-301 Rzeszów, Poland; mwarchalowska@o2.pl; 4 Orthopaedic and Rehabilitation Department, Medical University of Warsaw, 02-091 Warszawa, Poland; loleksy@oleksy-fizjoterapia.pl (Ł.O.); artur.stolarczyk@wum.edu.pl (A.S.); 5 Oleksy Medical & Sports Sciences, 37-100 Łańcut, Poland; 6 Department of Surgery with the Trauma and Orthopedic Division, District Hospital in Strzyżów, 38-100 Strzyżów, Poland

**Keywords:** conversion, laparoscopic cholecystectomy, open surgery, risk factors

## Abstract

Laparoscopic cholecystectomy is a standard treatment for cholelithiasis. In situations where laparoscopic cholecystectomy is dangerous, a surgeon may be forced to change from laparoscopy to an open procedure. Data from the literature shows that 2 to 15% of laparoscopic cholecystectomies are converted to open surgery during surgery for various reasons. The aim of this study was to identify the risk factors for the conversion of laparoscopic cholecystectomy to open surgery. A retrospective analysis of medical records and operation protocols was performed. The study group consisted of 263 patients who were converted into open surgery during laparoscopic surgery, and 264 randomly selected patients in the control group. Conversion risk factors were assessed using logistic regression analysis that modeled the probability of a certain event as a function of independent factors. Statistically significant factors in the regression model with all explanatory variables were age, emergency treatment, acute cholecystitis, peritoneal adhesions, chronic cholecystitis, and inflammatory infiltration. The use of predictive risk assessments or nomograms can be the most helpful tool for risk stratification in a clinical scenario. With such predictive tools, clinicians can optimize care based on the known risk factors for the conversion, and patients can be better informed about the risks of their surgery.

## 1. Introduction

Cholelithiasis is a serious problem in modern medicine. Gallbladder operations for cholelithiasis are the most common procedures performed in general surgery. Currently the majority of cholecystectomies are performed laparoscopically. Recent epidemiological studies indicate that there has been an increase in the incidence of gallstone disease in patients with coronary insufficiency and liver diseases [1,2].

Laparoscopic cholecystectomy (LC) is a standard treatment for gallstone disease [3,4,5]. LC results in a lower overall complication rate and shorter postoperative hospital stay compared to open cholecystectomy (OC) [6,7]. In situations where LC is dangerous, a surgeon may be forced to change from laparoscopy to the open procedure. Literature data shows that 2 to 15% of laparoscopic cholecystectomies are converted to open surgery during surgery for various reasons [8,9,10,11,12,13,14]. The most common causes are peritoneal adhesions and inflammatory infiltration of the gallbladder [11,12,13,15,16]. Converted cases are associated with an increased number of infectious and other postoperative complications [8,17,18,19,20], an increased risk of additional procedures, and a higher rate of readmission within 30 days [21]. Additionally, the conversion from laparoscopic to open surgery results in longer postoperative stays and higher morbidity and mortality rates in this group of patients [22].

Identifying preoperative patient-related factors, anticipating the need to convert from laparoscopic cholecystectomy to open surgery, can help identify high-risk patients and redefine surgical strategy in this group. These predictive conversion factors can also improve patient safety and increase the cost-effectiveness of gallstone treatment [23]. So far, the literature has shown many inconsistent factors that may result in the need to convert laparoscopy to open surgery in the treatment of gallstone disease. Among them, factors such as inflammatory infiltration, acute cholecystitis, age, sex, or coexisting diseases were found [4,8,24,25,26,27,28,29,30,31,32]. However, there are no reports on the time of day of laparoscopic cholecystectomy and its effect on the conversion to open procedure. Researchers indicate that time may play a role in the psychomotor performance of the doctor performing the surgery [33,34].

The aim of the study was to identify the risk factors for the conversion of laparoscopic cholecystectomy to open surgery. For this purpose, a retrospective assessment was made. The assessment involved some pre-operative factors that can predict the chances of conversion and the perioperative factors that led to conversion.

## 2. Materials and Methods

### 2.1. Study Design

It is a retrospective cohort study involving 527 from 3213 patients qualified for the treatment of cholecystolithiasis from the Department of General Surgery at the Provincial Clinical Hospital No. 2, of Saint Queen Jadwiga in Rzeszów (south-eastern Poland) over the period 2008–2018. A prior consent from the Director of the facility and the Head of the Department of Surgery for the use of the data has been obtained. The indication for cholecystectomy was symptomatic cholelithiasis. In all cases, the diagnosis of gallbladder stones was confirmed by ultrasound of the abdominal cavity. Basic laboratory examinations were performed in patients: blood group, blood count, urea, creatinine, electrolyte levels, fasting glucose, prothrombin time/international normalized ratio (INR/PT), activated partial thromboplastin time (APTT), bilirubin level, alkaline phosphatase, aspartate transaminase (AST), alanine transaminase (ALT), and thyroid stimulating hormone (TSH) were determined under the protocol in force in the hospital; all patients with symptomatic gallstones were initially qualified for laparoscopic surgery. Critically ill patients at high risk underwent open cholecystectomy and were excluded from the study. Patients who had cancer-induced cholecystectomy were also excluded from the study.

All operations were performed or supervised by the same team of surgeons who have been successfully performing this type of procedure in the hospital since 1992.

The consent to surgery included the patient’s consent to the laparotomy, if such a decision was made by the operating team during the laparoscopy. The procedures were performed in the operating theatre in compliance with all procedures as well as asepsis and antiseptics rules.

### 2.2. Surgical Procedure

Pneumoperitoneum is performed using a Veress needle at an intra-abdominal pressure ≤14 mmHg. Four trocars, two 5 mm and two 10 mm trocars, were carefully inserted into the abdominal cavity. The pressure of the peritoneal pneumoperitoneum was maintained in the range of 10 to 13 mmHg during the procedure. A standard set of cholecystectomy tools was used during the procedure. The Veress needle technique is the most commonly used, classical, and time-tested method. However, it is associated with very slow insufflation rates (depending on the brand of equipment) and potentially life-threatening complications [35]. Many studies evaluating the advantages and disadvantages of closed or open methods for creation of pneumoperitoneum have been conducted. However, randomized, multicenter clinical studies have not been able to provide a definite answer to which of the two methods is safer [36].

All patients underwent postoperative drainage of the peritoneal cavity using Redon’s method, maintained for 24 h, and with more drainage content for 2 to 3 days.

A retrospective analysis of electronic medical records and protocols of operations and examinations of 263 patients who had initially undergone laparoscopy and then converted to laparotomy were done. The remaining randomly selected patients (*n* = 264), who were not converted, were the control group. Acquiring data directly from the electronic database of medical records is the basis for proper data collection. The following factors were analyzed: sex; age; acute and chronic cholecystitis; time of operation, i.e., after 3 p.m., from 3 p.m. to 7 p.m., or after 7 p.m.; inflammatory infiltration; anatomical uncertainty; peritoneal adhesions; choledocholithiasis; the patient’s condition after ERCP (Endoscopic Retrograde Cholangiopancreatography); the patient’s condition after pancreatitis; acute surgery; and coexisting diseases (diabetes, hypertension, heart disease, and neurological diseases). Indications for the transition to open cholecystectomy were also reported.

### 2.3. Statistical Analyses

The data was collected in Microsoft Excel and analyzed using Statistica 13.1 (StatSoft, Inc., Tulsa, OK, United States). Statistical significance was established as a *p*-value less than 0.05. The characteristics of the patients who had their laparoscopy converted or non-converted to open surgery were compared using the Chi-Square test and the Mann–Whitney U test. The odds ratio (OR) and 95% confidence intervals (CIs) were calculated. Conversion risk factors were assessed using logistic regression analysis that modeled the probability of a certain event as a function of independent factors.

The quality of prediction of unplanned laparotomy based on individual regression models was assessed by calculating the following commonly applied parameters: Sensitivity or True Positive Rate (TPR) describes the ability to detect a disease in question and the Specificity or True Negative Rate (TNR) of people who do not have the disease—both of these measures take values from the range of 0% to 100%. Ideally, both of the values should be 100% in both tests.

PPV (Positive Predictive Value)—the percentage of patients with a positive test result who are actually in a distinguished health condition. NPV (Negative Predictive Value)—the percentage of patients with a negative result who are actually in a good health condition.

## 3. Results

### 3.1. Characteristics of the Study Group

Descriptive characteristics of the study sample are shown in Table 1 and Table 2.

The study group participants are significantly older (around 13 years), therefore age is a risk factor for the conversion to open surgery (Table 2).

There is also a difference in the gender structure of both groups as there are relatively fewer women in the study group than in the control group; therefore, the male gender is a risk factor for the conversion.

### 3.2. The Findings

#### 3.2.1. Analyses of Risk Factors for the Conversion

Table 3 presents the occurrence of the selected health factors considered as hypothetical risk factors for the conversion of laparoscopic cholecystectomy to open surgery. The majority of potential risk factors are significantly higher in the study group (*p* < 0.05).

Odds ratios for the influencing factors were calculated. The analysis also took into account age and gender; therefore, the risk factors were older age and male gender (Table 3).

#### 3.2.2. Multivariate Risk Analysis for the Conversion from Laparoscopic Cholecystectomy to Open Surgery

The following tables present the results after selecting the statistically significant risk factors using the forward stepwise regression procedure.

1. The early risk prediction model for the conversion from laparoscopy to open surgery.

In this model, information about the patient’s general condition (i.e., diabetes, high blood pressure, heart disease, and neurological diseases), age, and gender were taken into account (Table 4).

Statistically significant factors were age, sex, neurological diseases, and diabetes.

**Figure 1:**
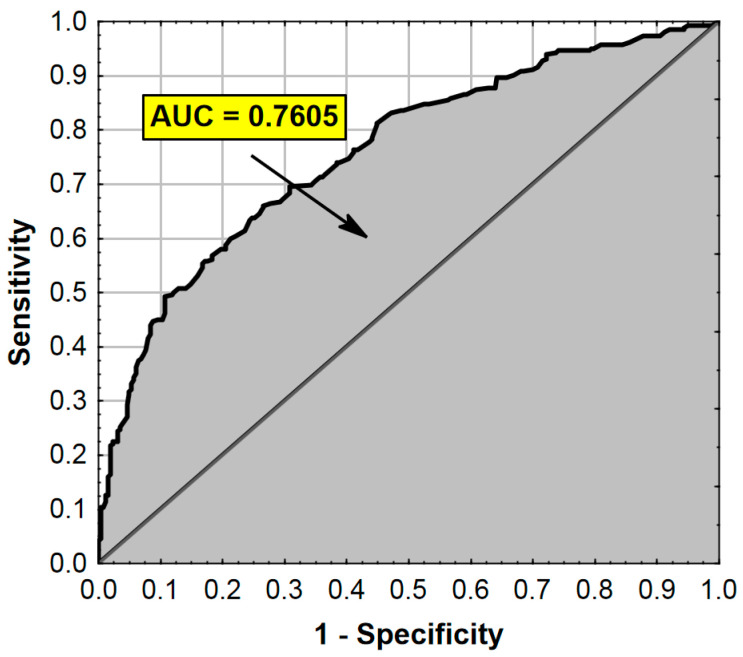
ROC curve and AUC value for the regression model from Table 4. AUC—Area Under the Curve (measure of diagnostic accuracy).

The ROC analysis (receiver operating characteristic curve) results are presented in Figure 1.

The logistic regression model formula to estimate the likelihood of the conversion from laparoscopy to open surgery for new patients is

*P* = exp(−3.2835 + 0.0480 × age + 0.8938 × gender + 1.6596 × neurological diseases + 0.6460 × diabetes),

*P* = 1 + exp(−3.2835 + 0.0480 × age + 0.8938 × gender + 1.6596 × neurological diseases + 0.6460 × diabetes).

The results of the classification patients in the studied population based on the logistic regression are presented in Table 5.

2. The perioperative factors model for the conversion of laparoscopy to open surgery.

In this model, information about the time (before 3 p.m., after 7 p.m.), mode of the procedure (emergency), and various types of inflammatory changes were taken into account: inflammatory infiltration, peritoneal adhesions, status after ERCP, and chronic and acute vesiculitis (Table 6).

**Figure 2:**
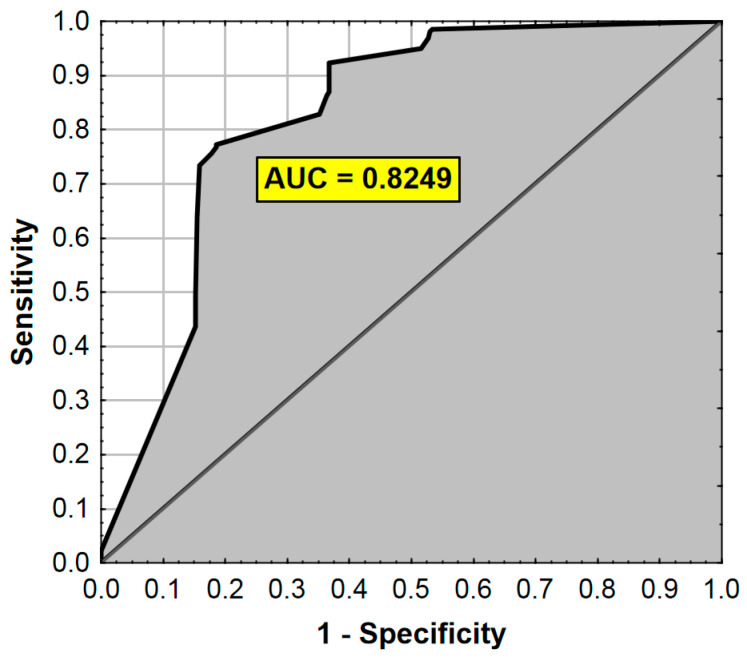
ROC curve and AUC value for the regression model from Table 6. AUC—Area Under the Curve (measure of diagnostic accuracy).

The ROC analysis results are presented in Figure 2.

The logistic regression model formula for the estimation of the probability of the conversion with perioperative factors is

*P* = exp (−2.3888 + 2.1391 × acute cholecystitis + 1.0284 × inflammatory infiltrate + 1.3478 × peritoneal adhesions + 0.9349 × emergency treatment + 1.2025 × chronic cholecystitis),

*P* = 1+exp (−2.3888 + 2.1391 × acute cholecystitis + 1.0284 × inflammatory infiltrate + 1.3478 × peritoneal adhesions + 0.9349 × emergency treatment + 1.2025 × chronic cholecystitis).

The results of the classification of patients in the studied population based on the constructed logistic regression model are also presented in Table 7.

3. The occurrence of the conversion from laparoscopy to open surgery for all the factors.

The model included age and the perioperative factors (Table 8).

**Figure 3:**
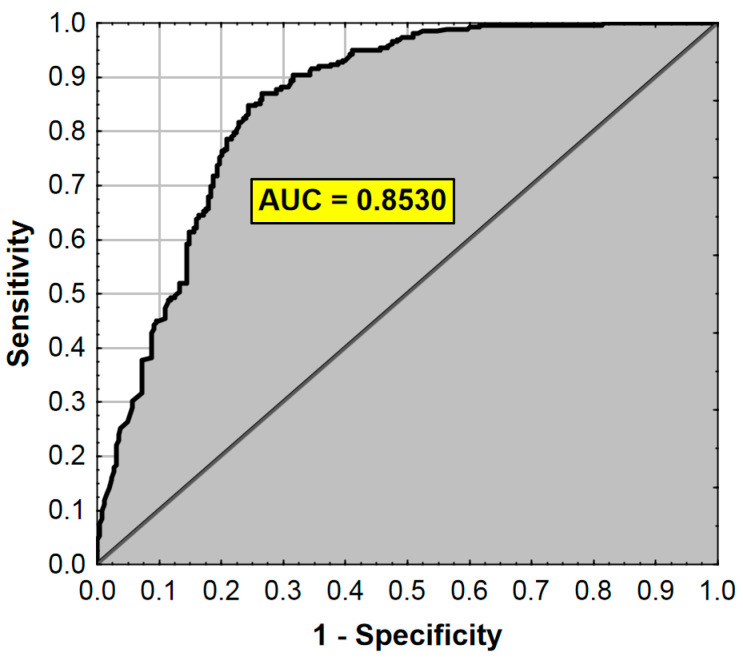
ROC curve and AUC value for the regression model from Table 8. AUC—Area Under the Curve (measure of diagnostic accuracy).

The ROC analysis results are presented in Figure 3.

The logistic regression model formula for the estimation of the probability of the conversion from laparoscopy to open surgery for all the factors is

*P* = exp (−4.1621 + 1.9967 × acute cholecystitis + 1.1541 × peritoneal adhesions + 0.0337 × age + 1.1396 × chronic cholecystitis + 0.8321 × emergency treatment + 0.8698 × inflammatory infiltrate),

*P* = 1 + exp (−4.1621 + 1.9967 × acute cholecystitis + 1.1541 × peritoneal adhesions + 0.0337 × age + 1.1396 × chronic cholecystitis + 0.8321 × emergency treatment + 0.8698 × inflammatory infiltrate).

The values of the diagnostic test quality measures using the regression model are at a satisfactory level (about 80%). They are very similar to the results obtained in Table 6 and Table 8. In this context, it is more correct to consider the probability of an emergency laparotomy separately on a perioperative basis than to combine it into a model that has almost the same predictive quality (Table 9).

## 4. Discussion

Laparoscopic cholecystectomy is currently considered as the gold standard in surgical treatment of gallbladder stones [37]. Many centers around the world use the LC more frequently than classical cholecystectomy (CC) due to low invasiveness and safety of the surgeries performed, reduction of postoperative complications, faster recovery, and significantly shorter hospital stay [38,39]. However, due to certain factors, there is occasionally a need to abandon the previously planned laparoscopic procedure and perform classic cholecystectomy [4,40].

The aim of the study was to retrospectively assess the reasons for the conversion of laparoscopic procedures to laparotomy at the Clinical Hospital No. 2 in Rzeszów over the period 2008–2018.

Analyzing the control and study group, it can be observed that age and sex of the respondents, similar as in the studies of other authors, constitute risk factors for unplanned laparotomy [5,41,42]. Taking into account the hypothetical risk factors for the occurrence of unplanned laparotomy, as presented in the paper, it has been shown that the time during the day when the surgery is performed is a statistically significant factor.

This applies to procedures performed after 3 p.m., when there is no full team of qualified and experienced surgeons in the hospital ward. Another reason may be the decrease in the psychomotor performance of surgeons, which decreases significantly with the passage of time during a working day, causing a lower efficiency and producing less-effective results of the surgical operations [43,44]. Other potential risk factors that are statistically significant for unplanned laparotomy include acute cholecystitis, choledocholithiasis, emergency surgery, diabetes, hypertension, heart disease, neurological disease, and, to a lesser extent, anatomical uncertainty. Factors such as chronic cholecystitis, peritoneal adhesions, patient’s status after ERCP, and status after pancreatitis were not statistically significant as potential conversion factors. In addition, other authors also took the following into account: patient’s BMI [45,46], thickness of the gallbladder wall [4,47], previous abdominal surgery [41,42], increased alkaline phosphatase activity and bilirubin levels [41,45], elevated white blood cell count [41,45,46], elevated body temperature, and the American Society of Anesthesiologists score above 3 [41,48]. Limited experience of a medical doctor performing LC is also considered as a statistically significant conversion factor [5,45].

The multivariate analysis carried out using the logistic regression method allowed us identify the factors responsible for the risk of unplanned laparotomy and to find the optimal model, useful for risk management during LC procedures. This can be used as the so-called early prediction model. Studies conducted in this area propose various point or predictive models regarding the likelihood of conversion [4,8,41]. Kama et al. proposed to assess the risk of conversion from laparoscopic to open cholecystectomy. Scores included such variables as male gender, abdominal tenderness, previous upper abdominal surgery, thickened gallbladder wall, aged over 60, and the presence of acute cholecystitis [4]. Lipman et al. developed an equation to predict the conversion based on statistically significant factors, namely, male sex, low serum albumin, elevated leukocytes, ultrasound pericholecystic fluid, diabetes, and elevated total bilirubin [25]. In turn, Goonawardena et al. developed a predictive model graphically illustrated with four probability nomograms that allows one to predict the conversion. The model used statistically significant variables, such as previous epigastric surgery, obesity, gallstone disease, thickening of the gallbladder wall, and a stone in the gallbladder neck [41].

The obtained results showed that the following were statistically significant: age (the chance of unplanned laparotomy increases 1.05 times every year), sex (in men the chance of an unplanned laparotomy is 2.44 times higher than in women), the occurrence of neurological diseases (the chance of an unplanned laparotomy is 5.26 times greater), and diabetes (1.9 times greater chance of unplanned laparotomy). Similar results in this respect were obtained by Masri et al. [37] and Coccolini et al., drawing conclusions from a meta-analysis carried out of the subject discussed [49]. Coelho, on the other hand, in studies assessing the role of gender in the results of surgery and the outcome of laparoscopic cholecystectomy, concludes that male gender is not an independent risk factor for laparoscopic conversion and perioperative complications. The researcher only points to a longer operation time in men (72.48 ± 28.50) than in women (65.46 ± 24.83, *p* < 0.001). The presented results may be a consequence of the unequal distribution of the studied groups of women and men (32.8% vs. 67.2%) [50].

Perioperative factors that influence the risk of unplanned laparotomy immediately before or during the procedure were also analyzed. The most important include acute cholecystitis, the presence of peritoneal adhesions, and chronic cystitis. Other researchers studying the factors responsible for the conversion of laparoscopic procedures to open surgery also propose a division into surgical factors, factors related to the patient’s health condition, related to the equipment used, and emphasize that the experience of the surgeon is also very important [4,51,52].

The use of the logistic regression model in the presented study allowed for the creation of a formula to estimate the probability of unplanned laparotomy in future patients. Identifying patients with significant conversion factors can significantly minimize the adverse effects of attempting laparoscopy. This can provide the basis for enabling hospitals to better plan treatments and efficiently manage their medical staff resources. Goonawardena et al., using multivariate logistic regression, created a model that may be a useful tool for hospitals to determine their own risk threshold. Such models are also useful from the patient’s point of view. They will help the patient to reasonably plan the time allocated for the surgery and related family and professional matters [41,42].

The study has several limitations. Firstly, there are slight differences in the homogeneity of the control group and the study group in terms of age and sex. Another limitation is the retrospective nature of the data collection (from the period 2008–2018), which may lead to a limited ability to correctly classify the preoperative diagnosis and makes the analyzed group very heterogeneous. Finally, this is a single-hospital study that limits the possibility of generalizing the results, and analyzing a random sample of successful laparoscopic cholecystectomies instead of the entire population is another limitation in itself. The formulae obtained from logistic regression should also be validated in the future on a different sample of respondents.

## 5. Conclusions

The conducted research revealed many significant risk factors related to conversion. LC is the surgery of choice for mild gallbladder disease. However, in the minority of LC patients, it will have to be converted to OS. The results are in line with previous studies that found male gender, old age, and comorbidities such as diabetes and neurological diseases to be the main risk factors for the conversion. The use of predictive risk assessments or nomograms can be the most helpful tool for risk stratification in a clinical scenario. With such predictive tools, clinicians can optimize care based on known risk factors for the conversion, and patients can be better informed about the risks of their surgery.

## Figures and Tables

**Table 1:** Characteristics of the studied and control groups by age.

Group	Age (Years) (*p* = 0.0000)						
	$\bar{x}$	Me	*s*	*C*_25_	*C*_75_	Min	Max
Control group (*n* = 264)	52.3	55	16.2	39	65	18	88
Study group (*n* = 263)	65.7	67	15.4	56	77	15	97

$\bar{x}$—arithmetic mean; Me—median; *s*—standard deviation; *C*_25_—the 25th percentile; *C*_75_—the 75th percentile; *p*—*p*-value, indicate significant values (*p* < 0.05); test probability values were calculated using the Mann–Whitney U test.

**Table 2:** Characteristics of the studied and control groups by gender.

Gender	Group (*p* = 0.0000)		Total
	Control Group	Study Group	
Female	200 (75.8%)	143 (54.4%)	343
Male	64 (24.2%)	120 (45.6%)	184
Total	264	263	527

*p*—*p*-value, indicate significant values (*p* < 0.05); test probability value was determined using the Chi-Square test of independence.

**Table 3:** Odds ratio values for the risk factors.

Risk Factors for Conversion	Group					*p*
	Control Group		Study Group			
	*n*	%	*n*	%	OR	
Male gender	64	24.2%	120	45.6%	2.62 (1.81–3.80)	0.0000
Age over 60	86	32.7%	177	67.6%	4.29 (2.97–6.17)	0.0000
Acute cholecystitis	49	18.6%	180	68.4%	9.52 (6.35–14.26)	0.0000
Chronic cholecystitis	51	19.3%	54	20.5%	1.08 (0.70–1.65)	0.7272
Time—before 3 p.m.	100	37.9%	129	49.1%	5.85 (3.79–9.03)	0.0000
Time—from 3 p.m. to 7 p.m.	87	33.0%	84	31.9%	4.70 (2.80–7.91)	0.0000
Time—after 7 p.m.	77	29.1%	50	19.0%	4.37 (2.20–8.70)	0.0000
Inflammatory infiltrate	87	33.0%	208	79.1%	7.69 (5.20–11.39)	0.0000
Anatomical ambiguity	0	0.0%	10	3.8%	×	0.0014
Peritoneal adhesions	41	15.5%	31	11.8%	0.73 (0.44–1.20)	0.2109
Choledocholithiasis	0	0.0%	11	4.2%	×	0.0008
State after ERCP	22	8.3%	27	10.3%	1.26 (0.70–2.27)	0.4449
State after inflammation of the pancreas	17	6.4%	9	3.4%	0.51 (0.23–1.18)	0.1098
Emergency treatment	48	18.2%	165	62.7%	7.58 (5.08–11.31)	0.0000
Diabetes	19	7.2%	45	17.1%	2.66 (1.51–4.69)	0.0005
Hypertension	51	19.3%	94	35.7%	2.32 (1.56–3.45)	0.0000
Heart diseases	15	5.7%	48	18.3%	3.71 (2.02–6.81)	0.0000
Neurological diseases	3	1.1%	18	6.8%	6.39 (1.86–21.97)	0.0008

ERCP—Endoscopic Retrograde Cholangiopancreatography. *p*—*p*-value, indicate significant values (*p* < 0.05); test probability values were calculated using the Chi-Square test of independence; *OR*—the odds ratio (with 95% confidence interval). ×—calculation of the odds ratio was impossible due to the lack of a risk factor in the control group.

**Table 4:** Statistical significance of the factors in the regression model with early risk factors.

Independent Variables	The Early Risk Prediction Model	
	OR (95% CI)	*p*
Age (years)	1.049 (1.036–1.063)	0.0000
Gender (male vs. female)	2.444 (1.628–3.671)	0.0000
Neurological diseases	5.257 (1.282–21.554)	0.0211
Diabetes	1.908 (1.035–3.517)	0.0384

CI—confidence interval; *p—p*-value, indicate significant values (*p* < 0.05).

**Table 5:** Results of the classification of patients in the studied population.

Classification Based on the Logistic Regression Model	Condition Observed		Total
	Study Group	Control Group	
Study group	176	78	254 (PPV = 69%)
Control group	86	185	271 (NPV = 68%)
Total	262 (TPR = 67%)	263 (TNR = 70%)	525

TPR—True Positive Rate; PPV—Positive Predictive Value; NPV—Negative Predictive Value.

**Table 6:** Statistical significance of the factors in the regression model with perioperative factors.

Independent Variables	The Perioperative Factors Model	
	OR (95% CI)	*p*
Emergency treatment	2.547 (1.532–4.234)	0.0003
Acute cholecystitis	8.492 (4.527–15.930)	0.0000
Inflammatory infiltrate	2.797 (1.481–5.281)	0.0015
Peritoneal adhesions	3.849 (1.898–7.805)	0.0002
Chronic cholecystitis	3.328 (1.691–6.552)	0.0005

CI—confidence interval; *p—p*-value, indicate significant values (*p* < 0.05).

**Table 7:** Results of the classification of patients in the studied population.

Classification Based on the Logistic Regression Model	Condition Observed		Total
	Study Group	Control Group	
Study group	202	49	251 (PPV = 80%)
Control group	61	215	276 (NPV = 78%)
Total	263 (TPR = 77%)	264 (TNR = 81%)	527

TPR—True Positive Rate; PPV—Positive Predictive Value; NPV—Negative Predictive Value.

**Table 8:** Statistical significance of the factors in the regression model with all explanatory variables.

Independent Variables	The Occurrence of Emergency Laparotomy	
	OR (95% CI)	*p*
Age	1.034 (1.019–1.049)	0.0000
Emergency treatment	2.298 (1.364–3.872)	0.0018
Acute cholecystitis	7.365 (3.863–14.039)	0.0000
Peritoneal adhesions	3.171 (1.525–6.594)	0.0020
Chronic cholecystitis	3.126 (1.562–6.252)	0.0013
Inflammatory infiltrate	2.386 (1.246–4.572)	0.0087

CI—confidence interval; *p—p*-value, indicate significant values (*p* < 0.05).

**Table 9:** Results of the classification of the patients in the studied population.

Classification Based on the Logistic Regression Model	Condition Observed		Total
	Study Group	Control Group	
Study group	215	62	277 (PPV = 78%)
Control group	47	201	248 (NPV = 81%)
Total	262 (TPR = 82%)	263 (TNR = 76%)	525

TPR—True Positive Rate; PPV—Positive Predictive Value; NPV—Negative Predictive Value.
